# Dietary restriction-resistant human tumors harboring the PIK3CA-activating mutation H1047R are sensitive to metformin

**DOI:** 10.18632/oncotarget.1234

**Published:** 2013-08-21

**Authors:** Sílvia Cufí, Bruna Corominas-Faja, Eugeni Lopez-Bonet, Rosa Bonavia, Sonia Pernas, Isabel álvarez López, Joan Dorca, Susana Martínez, Norberto Batista López, Severina Domínguez Fernández, Elisabet Cuyàs, Joana Visa, Esther Rodríguez-Gallego, Rosa Quirantes-Piné, Antonio Segura-Carretero, Jorge Joven, Begoña Martin-Castillo, Javier A. Menendez

**Affiliations:** ^1^ Metabolism & Cancer Group, Translational Research Laboratory, Catalan Institute of Oncology, Girona, Catalonia (Spain); ^2^ Girona Biomedical Research Institute (IDIBGi), Girona, Catalonia (Spain); ^3^ Department of Anatomical Pathology, Dr. Josep Trueta Hospital of Girona, Girona, Catalonia (Spain); ^4^ Animal Care Facility, Bellvitge Research Institute (IDIBELL), L'Hospitalet de Llobregat, Barcelona, Catalonia (Spain); ^5^ Department of Medical Oncology, Breast Unit, Catalan Institute of Oncology-Hospital Universitari de Bellvitge-Bellvitge Research Institute (IDIBELL), L'Hospitalet de Llobregat, Barcelona, Catalonia (Spain); ^6^ Medical Oncology Service, Hospital Donostia, Donostia-San Sebastián, Basque Country (Spain); ^7^ Department of Medical Oncology, Catalan Institute of Oncology, Girona, Catalonia (Spain); ^8^ Medical Oncology Department, Hospital de Mataró, Mataró, Barcelona, Catalonia (Spain); ^9^ Medical Oncology Service, Hospital Universitario de Canarias, La Laguna, Tenerife, Canary Islands (Spain); ^10^ Medical Oncology Service, Hospital de Txagorritxu, Vitoria-Gasteiz, Araba, Basque Conutry (Spain); ^11^ Unitat de Recerca Biomèdica (URB-CRB), Institut d'Investigació Sanitaria Pere i Virgili (IISPV), Universitat Rovira i Virgili, Reus, Catalonia (Spain); ^12^ Department of Analytical Chemistry, Faculty of Sciences, University of Granada, Granada (Spain); ^13^ Unit of Clinical Research, Catalan Institute of Oncology, Girona, Catalonia (Spain); ^14^ On behalf of the METTEN-01 Investigators (EudraClinicalTrial Number 2011-000490-30)

**Keywords:** Metformin, cancer, PI3K, PIK3CA mutations, dietary restriction, calorie restriction, rapamycin

## Abstract

Cancer cells expressing constitutively active phosphatidylinositol-3 kinase (PI3K) are proliferative regardless of the absence of insulin, and they form dietary restriction (DR)-resistant tumors *in vivo*. Because the binding of insulin to its receptors activates the PI3K/AKT/mammalian target of rapamycin (mTOR) signaling cascade, activating mutations in the *PIK3CA* oncogene may determine tumor response to DR-like pharmacological strategies targeting the insulin and mTOR pathways. The anti-diabetic drug metformin is a stereotypical DR mimetic that exerts its anti-cancer activity through a dual mechanism involving insulin-related (systemic) and mTOR-related (cell-autonomous) effects. However, it remains unclear whether *PIK3CA*-activating mutations might preclude the anti-cancer activity of metformin *in vivo*. To model the oncogenic *PIK3CA*-driven early stages of cancer, we used the clonal breast cancer cell line MCF10DCIS.com, which harbors the gain-of-function *H1047R* hot-spot mutation in the catalytic domain of the *PI3KCA* gene and has been shown to form DR-refractory xenotumors. To model *PIK3CA*-activating mutations in late stages of cancer, we took advantage of the isogenic conversion of a PIK3CA-wild-type tumor into a PIK3CA H1047R-mutated tumor using the highly metastatic colorectal cancer cell line SW48. MCF10DCIS.com xenotumors, although only modestly affected by treatment with oral metformin (approximately 40% tumor growth inhibition), were highly sensitive to the intraperitoneal (i.p.) administration of metformin, the anti-cancer activity of which increased in a time-dependent manner and reached >80% tumor growth inhibition by the end of the treatment. Metformin treatment via the i.p. route significantly reduced the proliferation factor mitotic activity index (MAI) and decreased tumor cellularity in MCF10DCIS.com cancer tissues. Whereas SW48-wild-type (PIK3CA+/+) cells rapidly formed metformin-refractory xenotumors in mice, ad libitum access to water containing metformin significantly reduced the growth of SW48-mutated (PIK3CAH1047R/+) xenotumors by approximately 50%. Thus, metformin can no longer be considered as a bona fide DR mimetic, at least in terms of anti-cancer activity, because tumors harboring the insulin-unresponsive, DR-resistant, PIK3CA-activating mutation H1047R remain sensitive to the anti-tumoral effects of the drug. Given the high prevalence of PIK3CA mutations in human carcinomas and the emerging role of PIK3CA mutation status in the treatment selection process, these findings might have a significant impact on the design of future trials evaluating the potential of combining metformin with targeted therapy.

## INTRODUCTION

The most hotly debated and yet unresolved issue in the area of employing calorie restriction (CR) mimetics (CRMs) as anti-cancer agents with mammalian target of rapamycin (mTOR)-inhibiting activity is the relative effects of nature *versus* nurture, i.e., the relative contributions of systemic factors *versus* cancer cell-autonomous effects [1-3]. The comparison of energy-balance regimens, such as CR, and pharmacological interventions with well-established mTOR inhibitors has revealed that rapamycin conspicuously mimics CR in its ability to decrease the mammary tumor burden in obese animals [4-6]. However, constitutively active mTOR has been found to fully ablate the beneficial effects of CR on mammary tumor growth [7]. Similarly, tumors with constitutively active phosphatidylinositol-3 kinase (PI3K) have been found to be refractory to the anti-cancer activity of dietary restriction (DR) [8, 9]. Because the binding of insulin to its receptors activates the PI3K/AKT pathway, which is known to stimulate mTOR activity, Kalaany and Sabatini's observations that tumor cells bearing constitutively activated PI3K mutations are proliferative *in vitro* in the absence of insulin or IGF-1 and form DR-resistant tumors *in vivo* [8] clearly suggest that cancer cell-autonomous alterations (e.g., activating mutations of PI3K) may ultimately determine the response of cancer cells to CR, DR, or CRMs.

One mTOR-inhibiting drug with great promise as an anti-cancer CRM is metformin [10-16], a biguanide drug that is commonly used to treat type 2 diabetes. Epidemiological studies have consistently suggested that type 2 diabetic patients treated with metformin have a lower risk of developing and dying from cancer than do diabetic patients receiving sulfonylurea, insulin, or other therapies [17, 18]. Because insulin resistance and consequent hyperinsulinemia can promote carcinogenesis directly through the insulin receptor or indirectly by increasing the levels of insulin-like growth factors or promoting persistently elevated plasma glucose, the association between metformin consumption and the reduced risk of cancer among type 2 diabetic patients may be explained simply by the metformin-driven improvement of insulin levels and blood glucose [19-22]. Indeed, the serum insulin- and IGF-1-lowering effects of metformin have been thought to explain why the administration of metformin suppresses tumor development or growth in multiple experimental models, including colon, hematopoietic, and mammary cancer models. However, decreases in hyperglycemia and *hyperinsulinemia* are not always correlated with the anti-cancer efficacy of metformin, as can be observed in non-diabetic mouse models. Thus, the anti-tumoral action of metformin is not precluded in *PTEN^+/−^* [23, 24], *HER2* [25, 26], and APC^min/+^ [27] mouse tumor models, in which insulin-related markers are not significantly attenuated by treatment with metformin. The notion that metformin suppresses cancer growth through pathways other than insulin/IGF-1-dependent, indirect drug action has recently been supported by experiments in liver IGF-1-deficient (LID) mice, which had naturally decreased lung tumor multiplicity and burden compared with wild-type mice [28, 29]. In this model, metformin further decreased tobacco carcinogen (NNK)-induced lung tumorigenesis without affecting IGF-1 levels. The authors demonstrated that metformin can act through IGF-1-independent mechanisms by promoting the systemic inhibition of circulating growth factors and local receptor tyrosine kinase signaling in cancer tissues [28]. Although these findings suggest an insulin-independent, direct anti-tumoral activity for metformin, it remains unknown whether cancer cell-autonomous alterations that are known to drive insulin/IGF-1-independent, DR-resistant tumors preclude the anti-cancer activity of metformin.

We recently proposed that CR/DR-unresponsive tumors with activating mutations in the *PIK3CA* oncogene should be monitored for their responsiveness to clinically relevant concentrations of metformin. We now present evidence that human cancer xenotumors harboring the insulin-unresponsive *PIK3CA*-activating mutation *H1047R* remain sensitive to metformin. Our data confirm that metformin cannot be considered a *bona fide* CRM, at least in terms of its anti-cancer activity. Given the high number of *PIK3CA* mutations in human cancer, this finding could have a significant impact on the design of metformin-based therapies that aim to influence both the early stages of tumor formation and progression and cancer recurrence in advanced tumors.

## RESULTS

PIK3CA H1047R-mutated, DR-resistant MCF10DCIS.com xenotumors are highly sensitive to intraperitoneally (i.p.) administered metformin. To model the oncogenic *PIK3CA*-driven early stages of human cancer, we employed the clonal breast cancer cell line MCF10DCIS.com, which was derived from a xenograft originating from premalignant MCF10AT cells that were injected into SCID mice. The MCF10DCIS.com cell line has been shown to have a missense mutation, *H1047R*, in the kinase domain of PI3K. This gain-of-function mutation is one of the “hot-spot” mutations in the catalytic domain p110α of the *PI3KCA* gene, and it generates the most potently oncogenic PI3K that is found with high frequency in various cancers. More importantly for this study, MCF10DCIS.com cells have been shown to proliferate regardless of the presence or absence of insulin *in vitro* and form tumors that are refractory to DR *in vivo* [8].

Two different metformin treatment protocols were tested (n=5 mice per group). In the first protocol, mice were exposed to control (water) or metformin (250 mg kg^−1^) *ad libitum* 1 week prior to tumor cell inoculation. The water was changed twice weekly and provided continuously for a total of 56 days. In the second protocol, metformin was administered by i.p. injection, and mice were treated daily with 200 mg kg^−1^ metformin beginning 1 week before cell inoculation. This i.p. schedule with metformin was continued for a total of 56 days. Metformin was well tolerated in both treatment regimens, and it did not significantly affect the weight (Supplementary Fig. 1) or the diet consumption profiles (data not shown) of the mice throughout the course of treatment.

**Figure 1:**
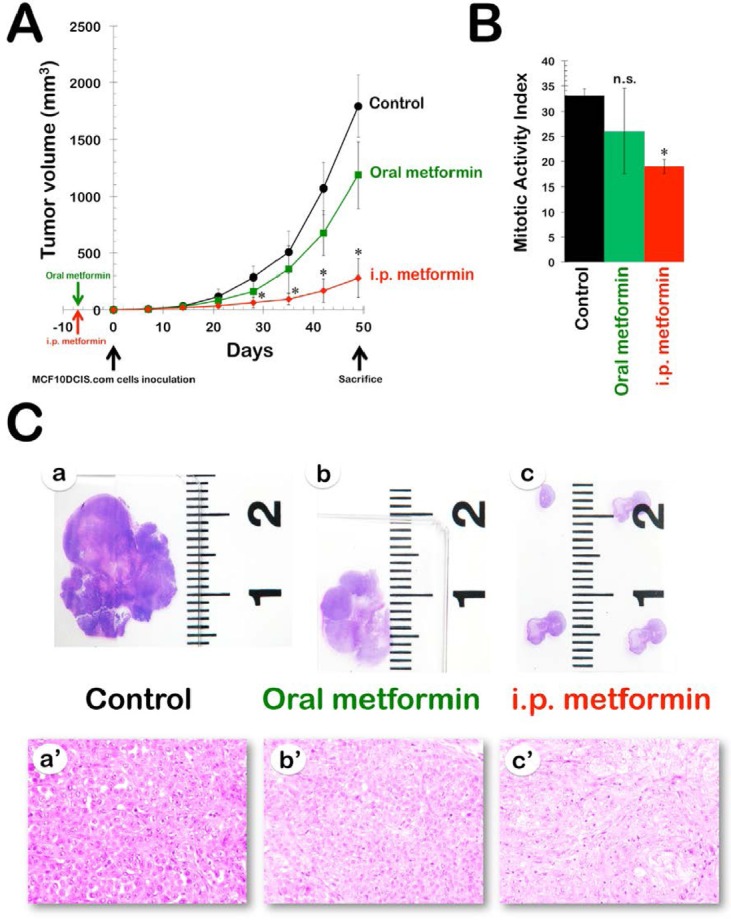
Efficacy of oral and i.p metformin in the DR-resistant MCF10DCIS.com xenograft model. A. Shown are the mean tumor volumes (±SD) of MCF10DCIS.com xenograft-bearing nude mice following oral (*ad libitum* access to water containing 250 mg kg^−1^ metformin) and i.p. (daily i.p. injections of 200 mg kg^−1^ metformin) administration of metformin for 8 weeks. Tumor growth rates were significantly different between the control and the i.p. metformin groups (* Student's *t*-test *P*<0.01). B. The bar graph (mean ± SD) shows the quantification of the mitotic activity in the xenografts. The number of mitosis per high power field was quantified by light microscopy in at least ten high power fields per tumor in all xenografts (n=2 per group, three groups; * Student's *t*-test *P*<0.01 versus control group). C. Metformin-treated MCF10DCIS.com xenotumors have reduced tumor growth and altered histological features. *a-c*. Sections from xenografts were stained with H&E and taken at low magnification. *a'-c'*. Histopathological comparison among MCF10DCIS.com xenografts (evaluation was performed under 400X objective magnification)

Efficacy of oral metformin in the DR-resistant MCF10DCIS.com xenograft model. Fig. 1A shows the rate of tumor growth in the three treatment groups, with the data plotted as the mean tumor volume in each group over time. Compared with the animals in the vehicle-treated group (mean xenografted MCF10DCIS.com tumor volume of 1794±273 mm^3^), the animals that received 8 weeks of treatment with oral metformin exhibited slightly decelerated tumor growth, with a final mean tumor volume of 1186±293 mm^3^ (Fig. 1A). However, none of the differences between the untreated controls and the oral metformin-treated xenotumors over time reached statistical significance. This finding was better reflected by the percent MCF10DCIS.com tumor growth inhibition, which was calculated as follows: 1-treated/control volume ratio (1-T/C). The inhibitory effect of oral metformin was modest, reaching a maximum of 43% at 4 weeks after cell inoculation and decreasing toward the end of the treatment (approximately 30-35%).

Efficacy of i.p. metformin in the DR-resistant MCF10DCIS.com xenograft model. We investigated whether the i.p. dosing schedule with metformin was more effective in preventing the growth of MCF10DCIS.com xenotumors and found that i.p. metformin was highly effective. Compared with the mean xenograft tumor volume (approximately 1800 mm^3^) in the untreated control animals, daily i.p. treatment with metformin resulted in a dramatic reduction in mean tumor volume to 280±171 mm^3^ (Fig. 1A). Notably, the anti-tumor activity of i.p. metformin at only 7 days after cell inoculation (49%) was already greater than the maximum activity achieved at any time with oral metformin. Moreover, the inhibitory effect of i.p metformin increased in a time-dependent manner, reaching a maximum of 84% at 42 days after cell inoculation.

Intraperitoneally administered metformin significantly decreases mitotic activity in MCF10DCIS.com cancer tissues. We analyzed MCF10DCIS.com tumor xenografts to investigate the potential proapoptotic and/ or anti-proliferative effects of metformin; these effects may have played a role in the overall anti-tumor efficacy of the drug. No significant differences were observed in the number of apoptotic cells between control tumors and the tumors from the two therapeutic regimens (i.e., mice treated with oral metformin and mice treated with i.p. metformin). We then compared the mitotic counts in hematoxylin and eosin (H&E)-stained paraffin-embedded sections according to the Scarff-Bloom-Richardson histoprognostic grading system, as modified by Elston and Ellis (Fig. 1B). For the first protocol, microscopic analysis of the H&E staining of MCF10DCIS.com tumors showed a moderate decrease (21%) in average mitotic counts in the oral metformin group (26±8) compared with the untreated control (33±1), but this difference did not reach statistical significance (p=0.5). For the second protocol, however, the quantification of average mitotic counts revealed a statistically significant (p=0.01) 42% decrease in the mitotic activity index in the i.p. metformin group (19±1) compared with the control group.

The microscopic appearance of MCF10DCIS.com carcinomas changed slightly after metformin treatment. Histological examination confirmed that the untreated MCF10DCIS.com tumors consisted of cells that appeared cytologically malignant, with high-grade nuclei, pleomorphism, and abundant central necrosis (i.e., Grade 3 invasive ductal carcinomas; Fig. 1C, panels a and a'). Anatomopathological examination of MCF10DCIS.com breast xenotumors in animals treated with oral metformin revealed that the cancer tissues remained poorly differentiated, despite visual evidence of a reduction in tumor size (Fig. 1C, panels b and b'). Interestingly, in the very small MCF10DCIS.com tumors that developed in the presence of daily i.p. metformin, a significant trend of decreasing cellularity, accompanied by an increase in the amount of extracellular connective tissue matrix, was observed (Fig. 1C, panels c and c').

**Figure 2:**
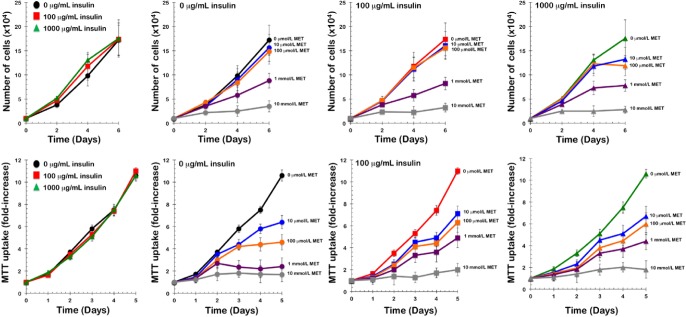
Metformin inhibits insulin-independent growth of PIK3CA-mutated tumor cells in vitro A. *Top*. Proliferation curves of MCF10DCIS.com cells cultured in the presence of increasing concentrations of insulin and/or metformin. MCF10DCIS.com cells were plated in 24-well plates at a density of 5,000 cells/well and cultured in 0.1% horse serum in the absence or presence of insulin (0, 100 and 1,000 ng/mL), metformin (1 μmol/L, 10 μmol/L, 100 μmol/L, 1 mmol/L, and 10 mmol/L), or a combination of insulin and metformin as specified. The data presented are the means of number cells × 10^4^/well (±SD) from one representative experiment made in triplicate and obtained after 0, 2, 4, and 6 days. *Bottom*. MTT uptake curves of MCF10DCIS.com cells cultured in the presence of increasing concentrations of insulin and/or metformin. MCF10DCIS.com cells were plated in 96-well plates at a density of ∼2,000 cells/ well and cultured in 0.1% horse serum in the absence or presence of insulin (0, 100 and 1,000 ng/mL), metformin (1 μmol/L, 10 μmol/L, 100 μmol/L, 1 mmol/L, and 10 mmol/L), or a combination of insulin and metformin as specified. The data presented are the means ±SD of fold-increases in OD_570_.

Insulin-independent growth of PIK3CA H1047R-mutated tumor cells is inhibited by metformin in vitro. Because the constitutive activation of the PI3K pathway drives MCF10DCIS.com cells to form DR-resistant tumors *in vivo* and to grow in an insulin-independent manner *in vitro*, we determined whether exogenous supplementation with metformin significantly affects tumor cell unresponsiveness to insulin *in vitro*. DR-resistant MCF10DCIS.com cells grew similarly in culture in an insulin-independent fashion, *i.e.*, insulin failed to cause a dose-dependent increase in cell number. Metformin did not act as an insulin sensitizer for insulin-resistant *PIK3CA H1047R*-mutated MCF10DCIS.com cells; rather, it acted as a growth inhibitor in a dose-dependent manner (Fig. 2).

**Figure 3:**
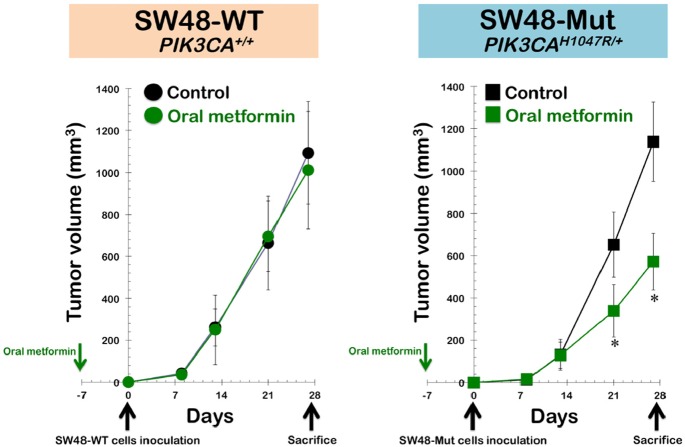
Isogenic conversion of a PIK3CA-wild-type tumor into a PIK3CA H1047R-mutated tumor promotes sensitization to the anti-cancer effects of metformin. Shown are the mean tumor volumes (±SD) of SW48-WT (*left panel*) and SW48-Mut (*right panel*) xenograft-bearing nude mice following oral (*ad libitum* access to water containing 250 mg kg^−1^ metformin) administration of metformin for ∼5 weeks. Tumor growth rates were significantly different between the control and the oral metformin groups in SW48-Mut xenografts (* Student's *t*-test *P*<0.01).

Isogenic conversion of a PIK3CA-wild-type tumor into a PIK3CA H1047R-mutated tumor promotes sensitization to the anti-cancer effects of metformin. To model *PIK3CA*-activating mutations in the late stages of human cancer, we used two types of cancer cells derived from the highly metastatic SW48 colorectal cancer cell line. These cell lines were isogenic, except that one line (SW48-WT) carries a wild-type allele of the *PIK3CA* oncogene, and the other (SW48-Mut) carries a constitutively active mutant allele (*H1047R*). In xenograft studies, the subcutaneous injection of SW48-WT or SW48-Mut cells into nude mice yielded tumors of approximately equal volume within 27 days (Fig. 3, *left panel*). The administration of an oral regimen of metformin to xenografted mice beginning 1 week before tumor cell injection failed to significantly alter tumor size in SW48-WT xenotumors over time. In contrast, SW48-Mut cells formed metformin-sensitive tumors that showed significant decreases in tumor volume under the metformin feeding condition. Compared with the mean xenograft tumor volume (1138±188 mm^3^) in the untreated control animals, oral treatment with metformin resulted in a notable reduction in the mean tumor volume, to 572±134 mm^3^ (Fig. 3, *right panel*). Indeed, the inhibitory effect of oral metformin against SW48-Mut xenotumors increased in a time-dependent manner, reaching a maximum of approximately 50% at 21 days after cell inoculation.

## DISCUSSION

The metabolism of tumors deviates significantly from that of corresponding normal cells and tissues [30-37]. Accordingly, the therapeutic use of DR/CR-mimetic agents to exploit the differential susceptibility of malignant *versus* normal cells to achieve energy metabolism inhibition is attracting great interest, and promising results have been obtained from cultured cells, animal models, and human trials. Although drugs targeting the cancer metabolic phenotype are expected to be “magic bullets” because many cancers show similar metabolic characteristics, it is important to note that molecular markers that can distinguish between CRM-sensitive and CRM-resistant tumors are lacking.

When searching for a cellular mechanism to explain the effect of DR on tumors, Kalaany and Sabatini found that DR reduces the activity of insulin-mediated signaling [8]. This signaling involves the activation of cell-surface receptors by insulin and the triggering of a downstream signaling cascade in the cell. This cascade is mediated by the enzyme PI3K and its suppression by DR enhances cell death and reduces tumor size in several, but not all, types of tumor cells. Cancer cell lines with mutations that cause the constitutive activation of PI3K (or loss-of-function mutations in PTEN, which normally antagonizes PI3K) are fully refractory to the tumor growth-inhibitory effects of DR. These findings are consistent with the effects of DR on the growth of early tumors due to both systemic changes in the host (e.g., circulating levels of insulin or IGF-1) and signaling events that are intrinsic to the tumor cells (e.g., those involving AMPK, mTOR, or SIRT1, all of which interact with the PI3K pathway at multiple levels). In this scenario, the pivotal involvement of the insulin-mediated pathway in determining the ability of DR to induce anti-tumor effects supports the hypothesis that drugs that ameliorate insulin resistance in type 2 diabetes might be beneficial in preventing cancer, even in non-diabetic patients. The same model, however, can be used to predict which tumors would be vulnerable to such DR-mimicking pharmacological treatments based on their mutation profiles, particularly in the genes encoding *PI3K* and *PTEN*. Because it is now evident that cancers of the liver, colon, and breast are the most likely to harbor *PIK3CA* mutations [38-44], with average mutational frequencies (across reported studies) of 36%, 26%, and 25%, respectively, a lack of responsiveness of *PI3K*-mutated carcinomas to putative DR mimetics such as metformin could have a substantial impact on the ever-growing number of randomized trials that are underway to evaluate whether and how metformin influences both the early stages of tumor formation and progression and cancer recurrence in advanced tumors [45-50].

Here, for the first time, we reveal that *PIK3CA* *H1047R*-mutated, DR-resistant MCF10DCIS.com xenotumors are highly sensitive (>80% tumor growth inhibition) to the daily i.p. administration of metformin. Because the oral dosing schedule of metformin only modestly affected tumor growth (approximately 40% of tumor growth inhibition), it is tempting to suggest that i.p. injections of metformin provoke peak plasma concentrations that are higher than the levels achieved with oral administration [29]. Pharmacokinetic analyses are currently underway to confirm whether the i.p. administration of metformin produces higher plasma levels of metformin, which might cause more significant anti-tumor effects. Nevertheless, there was a correlation between the metformin-stimulated decrease in proliferation factor mitotic activity index (MAI) in orally or intraperitoneally treated tumors and the overall efficacy of the route of metformin administration in preventing tumor growth in DR-resistant MCF10DCIS.com xenotumors. Because the significant decrease in the number of mitotic figures in tumors treated with i.p. metformin compared with untreated controls was not coupled with a change in the number of apoptotic cells, these findings suggested that the antitumor activity of i.p. metformin against MCF10DCIS.com breast cancer tumor growth mainly involved the inhibition of cell proliferation. Moreover, tumors treated with i.p. metformin showed a relatively substantial increase in stromal connective tissue that was not supportive of tumor growth [51], as this increase was accompanied by a decrease in mitotic figure numbers and reduced cellularity in the small amount of tumor tissue that remained at the end of the treatment. Proliferation factor MAI is the strongest prognostic factor in early breast cancer [52-54], a loss of cellularity has been shown to correlate with better prognosis and clinical outcome [55-57], and, ideally, a combination of residual tumor size and changes in tumor cellularity is useful in documenting treatment response and outcome. For these reasons, the response of *PIK3CA H1047R*-mutated breast cancer xenotumors to the anti-cancer effects elicited by the i.p. administration of metformin might be predictive of more favorable patient outcomes in a clinical setting. It would be interesting to investigate whether longer i.p. metformin regimens could still suppress *PIK3CA H1047R*-mutated tumors or whether tumors recur after some interval. Future studies should elucidate whether responsive tumors harboring *PI3K*-activating mutations will become resistant to the beneficial effects of metformin with time, as most tumors acquire resistance to chemotherapy.

At advanced tumor stages, signaling pathways other than the PI3K pathway have been suggested to play key roles in mediating the anti-cancer effects of DR. Hence, DR may no longer modulate signaling in advanced tumors once these tumors activate growth and survival pathways in a growth factor-independent fashion. This study is the first to reveal that, remarkably, the isogenic conversion of a *PIK3CA*-wild-type tumor into a *PIK3CA H1047R*-mutated tumor is sufficient to promote sensitization to the anti-cancer activity of metformin in an *in vivo* model of metastatic colon cancer. Considering the emerging role of *PIK3CA* mutation status in the treatment selection process [58-60], these findings might have a significant impact on the design of future trials evaluating the potential of combining metformin with targeted therapy. The hyperactivation of PI3K signaling downstream of the tyrosine kinase receptor HER2, either through loss-of-function *PTEN* mutations or dominant activating mutations in the catalytic subunit of PI3K, has been shown to significantly impair the efficacy of HER2-targeted drugs such as trastuzumab [61-68]. Accordingly, PI3K inhibitors, mTOR inhibitors, and dual PI3K/mTOR inhibitors are being evaluated as effective therapies for overcoming trastuzumab resistance. In this regard, it is interesting that the JIMT-1 breast cancer cell line, a model of intrinsic resistance to trastuzumab that harbors an activating mutation in the *PIK3CA* gene and low expression of PTEN [69], was notably responsive to metformin *in vitro* and *in vivo* [70, 71]. Moreover, when trastuzumab was combined with metformin to treat *PIK3CA*-mutated HER2^+^ breast carcinomas, tumor volume decreased sharply, thus suggesting that metformin is sufficient to overcome *in vivo* primary resistance to trastuzumab in these tumors [71]. Because of the accumulating evidence that *PIK3CA* mutation/PTEN expression status predicts colon carcinoma response to the EGFR inhibitor cetuximab [72-74], we are currently investigating whether the combination of cetuximab with metformin could be an effective therapeutic option in *PIK3CA*-mutated colon cancer.

After the *TP53* suppressor gene, *PIK3CA* is one of the most frequently mutated (gain-of-function) genes in several human carcinomas. A landmark study by Buzzai *et al.* [75] used the paired isogenic colon cancer cell lines HCT116 p53^+/+^ and HCT116 p53^−/−^ to test systemic treatment with metformin (250 mg kg^−1^ administered by daily i.p. injections). Using the clonal breast cancer cell line MCF10DCIS.com, which harbors the gain-of-function *H1047R* hot-spot mutation in the catalytic domain of the *PI3KCA* gene and has been shown to form DR-refractory xenotumors [8], and taking advantage of the isogenic conversion of a wild-type tumor into a *PIK3CA H1047R*-mutated tumor using the highly metastatic SW48 colorectal cancer cell line, we have shown that metformin can no longer be considered a *bona fide* DR mimetic, at least in terms of anti-cancer activity. In particular, tumors harboring the insulin-unresponsive, DR-resistant, *PIK3CA*-activating mutation *H1047R* remain sensitive to the anti-tumoral effects of the drug. Given the high prevalence of *PIK3CA* mutations in human carcinomas, and considering the emerging role of *PIK3CA* mutation status in the treatment selection process, these findings may have a significant impact on the design of future trials evaluating the potential of combining metformin with targeted therapy.

## METHODS

### Cell lines

MCF10DCIS.COM cells were purchased from Asterand, Inc. (Detroit, MI, USA). X-MAN^™^ isogenic cell lines were obtained from Horizon Discovery Ltd (http://www.horizondiscovery.com). The X-MAN^™^ isogenic cell line SW48 PI3Kα (H1047R/+), heterozygous knock-in of PIK3CA kinase domain activating mutation, was used in this study (HD103-005). The parental cell line, SW48 PI3K (+/+) was also used. Cells were maintained according to the supplier recommendations.

### Tumor xenograft study

Mice were randomly divided into groups of five mice each and orally or intraperitoneally treated as described in the Results section. To produce xenografts, approximately 5 × 10^6^ cells were subcutaneously injected into the dorsal flanks of female athymic nude mice (4-5 weeks old, 23-25 g; Harlan Laboratories). In both treatment regimens, body weight and diet consumption was determined weekly after dosing; tumor size was measured daily with electronic calipers; and tumor volume was calculated using the following formula: volume (mm^3^) = length × width^2^ × 0.5. The experiments were approved by the Institutional Animal Care and Use Committee (IACUC) of the Institut d'Investigació Biomèdica de Bellvitge (IDIBELL; Animal Use Protocol #6302 authorized by the Animal Experimental Commission of the Catalan Government, Barcelona, Spain). After 56 days of metformin treatment, the mice were euthanized by cervical dislocation. The tumors were excised and weighed, and one portion of the tissue was fixed in buffered formalin, whereas the remaining portion was stored at −80°C for further analysis. Tumor volumes were compared using Student's *t*-test. At the end of the tumorigenesis studies, plasma was isolated by centrifugation of blood samples, and the levels of circulating insulin were measured using the Rat/Mouse Insulin ELISA Kit (Millipore, Billerica, MA).

### Histology

At the appropriate time points, animals were euthanized, and tumors were removed and cut in half. One half was fixed in 10% buffered formalin, and the other was snap frozen. To assess apoptosis and mitosis in the tumor tissues, sections from formalin-fixed, paraffin-embedded xenograft tissues were stained with H&E.

### MAI

The mitotic figures observable following the H&E staining protocol were defined according to van Diest *et al.* [52], with some modifications, as follows: a) the absence of nuclear membrane signifying the end of prophase, and b) the presence of condensed chromosomes that were clustered together (beginning metaphase), arranged in a plane (metaphase or anaphase), or in separate clusters (telophase) all counted as one mitotic figure. Hyperchromatic nuclei, fragmented chromatin, and apoptotic nuclei were ignored. All H&E-stained sections were examined carefully (magnification, ×400), and the section showing the highest proliferation was selected for an assessment of mitotic activity. Starting from the subjectively most mitotically active area of the tumor and moving between consecutive fields, approximately 10 consecutive high-power fields (HPFs) were counted with an Olympus BH-2 microscope. No attempts were made to maximize counting by selecting those fields with a greater number of mitotic figures. Mitotic counts were performed without the knowledge of the treatment group-based immunohistochemical staining. MAI activities were compared using a two-tailed, two-sample, equal-variance Student's *t*-test.

### Proliferation assay

On day 0, cell lines were seeded in the appropriate media in 24-well plates at a density of 5,000 cells/well, and all plates were incubated overnight. On day 1, the assay plates for each cell line were washed twice with regular medium in the absence of serum, and the medium was replaced with medium supplemented with 0.1% serum alone, 0.1% serum and one of two different concentrations of insulin (100 and 1,000 ng/mL), 0.1% serum and one of seven different concentrations of metformin (10 nmol/L, 100 nmol/L, 1 μmol/L, 10 μmol/L, 100 μmol/L, 1 mmol/L, and 10 mmol/L), or a combination of insulin and metformin, as specified. Three wells per media condition were included in each plate. An additional plate for each cell line was used as a baseline day 0 measurement of cell number without addition of the assay media. Cell numbers were counted at days 0, 2, 4, and 6 using a Coulter Counter (Coulter Electronics, Inc.).

### Cell viability assays

The effect of metformin on cell viability in the absence or presence of insulin was determined using a standard colorimetric 3,4,5-dimethylthiazol-2-yl−2,5-diphenyl-tetrazolium bromide (MTT) reduction assay. For each treatment, the percent cell viability was calculated using the following equation: (OD_570_ of the treated sample/OD_570_ of the untreated sample) × 100.

## Collaborators (METTEN-01 Investigators):

Maria Luque Cabal (Medical Oncology Service, Hospital Central de Asturias, Oviedo, Spain), César A. Rodríguez-Sánchez (Medical Oncology Service, Hospital Universitario de Salamanca, Salamanca, Spain), Jose Luis Alonso Romero (Department of Medical Oncology, Hospital Universitario Virgen de la Arrixaca, Murcia, Spain), Ricardo Cubedo Cervera (Department of Medical Oncology, Hospital Puerta de Hierro, Madrid, Spain), Jose Manuel López-Vega (Medical Oncology Department, Hospital Universitario Marqués de Valdecilla, Santander, Spain), Noelia Martínez Jáñez (Medical Oncology Department, Hospital Universitario Ramón y Cajal, Madrid, Spain), Kepa Amillano Parraga (Medical Oncology Service, Hospital Universitario San Joan de Reus, Reus, Spain).
